# Heparanase Affects Food Intake and Regulates Energy Balance in Mice

**DOI:** 10.1371/journal.pone.0034313

**Published:** 2012-03-27

**Authors:** Linda Karlsson-Lindahl, Linnéa Schmidt, David Haage, Caroline Hansson, Magdalena Taube, Emil Egeciouglu, Ying-xia Tan, Therese Admyre, John-Olov Jansson, Israel Vlodavsky, Jin-Ping Li, Ulf Lindahl, Suzanne L. Dickson

**Affiliations:** 1 Institute of Neuroscience and Physiology/Endocrinology, the Sahlgrenska Academy at the University of Gothenburg, Gothenburg, Sweden; 2 Department of Integrative Medical Biology, Section for Physiology, Umeå University, Umeå, Sweden; 3 AstraZeneca, R&D Mölndal, Sweden; 4 Cancer and Vascular Biology Research Center, the Bruce Rappaport Faculty of Medicine, Technion, Haifa, Israel; 5 Department of Medical Biochemistry and Microbiology, University of Uppsala, Uppsala, Sweden; Nathan Kline Institute and New York University School of Medicine, United States of America

## Abstract

Mutation of the melanocortin-receptor 4 (MC4R) is the most frequent cause of severe obesity in humans. Binding of agouti-related peptide (AgRP) to MC4R involves the co-receptor syndecan-3, a heparan sulfate proteoglycan. The proteoglycan can be structurally modified by the enzyme heparanase. Here we tested the hypothesis that heparanase plays a role in food intake behaviour and energy balance regulation by analysing body weight, body composition and food intake in genetically modified mice that either lack or overexpress heparanase. We also assessed food intake and body weight following acute central intracerebroventricular administration of heparanase; such treatment reduced food intake in wildtype mice, an effect that was abolished in mice lacking MC4R. By contrast, heparanase knockout mice on a high-fat diet showed increased food intake and maturity-onset obesity, with up to a 40% increase in body fat. Mice overexpressing heparanase displayed essentially the opposite phenotypes, with a reduced fat mass. These results implicate heparanase in energy balance control via the central melanocortin system. Our data indicate that heparanase acts as a negative modulator of AgRP signaling at MC4R, through cleavage of heparan sulfate chains presumably linked to syndecan-3.

## Introduction

Obesity and overweight continue to increase world-wide, with severe health consequences that pose heavy economic burdens on society [1]. The balance between food intake and energy expenditure is monitored and tightly regulated by the central nervous system. The hypothalamus integrates afferent information from metabolic and hunger/satiety signals with diverse brain circuits involved in ingestive behaviour as well as energy expenditure. Within the hypothalamus, the melanocortin system represents a crucial point of convergence for these signals and is therefore of primary importance for the regulation of body weight [2], [3], [4]. The melanocortin receptor ligands, α-melanocyte stimulating hormone (α-MSH) and agouti-related peptide (AgRP), modulate downstream homeostatic signaling via their action at melanocortin receptors MC3R and MC4R. The agonist α-MSH reduces appetite and increases energy expenditure [4]. These effects are opposed by AgRP, an obesity-inducing peptide that operates as an inverse agonist at MC4R *in vitro* and *in vivo* [5]. Mice and humans that lack α-MSH or have defective MC4R signaling are hyperphagic, obese and show increased linear growth [6], [7], [8]. Conversely, ablation of AgRP neurons in adult mice causes acute anorexia [9] whereas mice that overexpress AgRP become obese [10]. AgRP deficient mice have a more subtle energy balance disturbance with a late-onset lean phenotype [11]. In humans, a defective MC4R is the most common cause of inherited severe obesity [12].

The binding of AgRP to MC4R is facilitated by syndecan-3, a heparan sulfate proteoglycan (HSPG) that appears to operate as a co-receptor [13]. The HS polysaccharide side chains of HSPGs provide binding epitopes for numerous proteins, and thus serve as co-receptors for various ligands to enable fine-tuning of signals [14], [15]. Importantly, HS was found to directly bind AgRP but not α-MSH [16]. Hypothalamic syndecan-3 has been implicated in energy balance regulation [13], [16], [17], [18] and is up-regulated during fasting [16]. Indeed, mice that lack syndecan-3 have a reduced fat mass and are protected against diet-induced obesity [17].

Heparanase selectively cleaves HS chains at glucuronidic linkages [19], and thus affects a variety of physiological and pathophysiological processes that depend on HS-mediated cell signalling [20]. Inspired by the potential importance of syndecan-3 HSPG in central melanocortin signaling [20] we sought to determine whether heparanase has a role in energy balance. Our studies explored genetic mouse models of altered heparanase expression that either lack [21] or overexpress [22] heparanase, along with experiments in which recombinant heparanase was administered into the brain ventricles. The results indicate that heparanase inhibits obesity by degrading HS chains, presumably linked to syndecan-3, thereby suppressing the binding of AgRP to MC4R.

## Results

### Heparanase knockout mice develop maturity-onset obesity and have altered feeding responses

**Figure 1:**
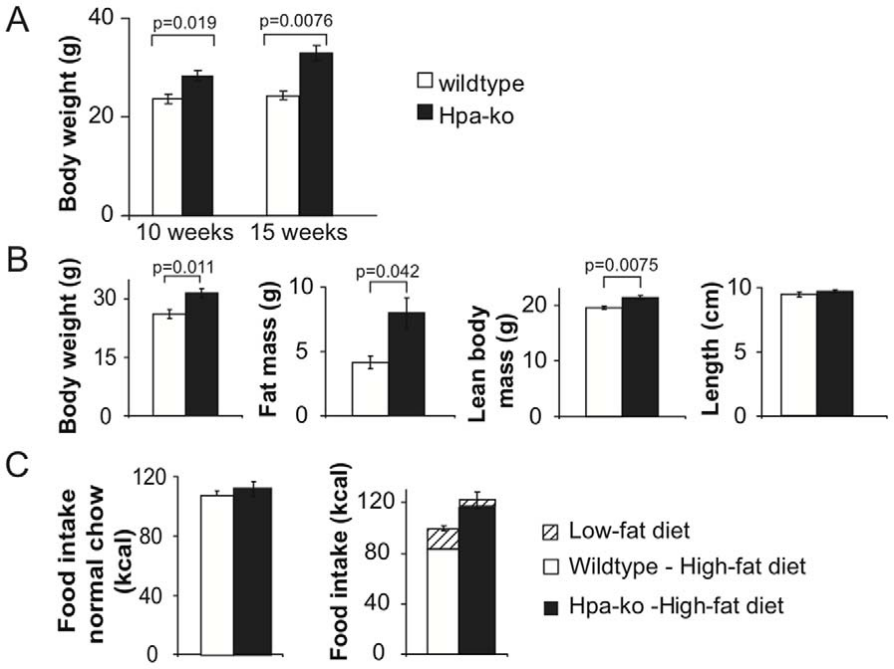
Increased body weight, altered body composition and food intake of *Hpa*-ko mice. (A) Increased body weight at maturity onset of *Hpa*-ko mice. Male *Hpa*-ko mice kept on normal chow showed increased body weight compared to wt sibling controls, discernible from 10 weeks of age (wt, n = 7; *Hpa-*ko, n = 20) and more pronounced at 15 weeks of age (wt, n = 5; *Hpa-*ko, n = 17). (B) Increased fat mass and lean body mass of *Hpa*-ko mice. DEXA analysis of 21-week old animals showed larger fat mass and lean body mass in *Hpa*-ko mice (n = 7) than in controls (n = 4). There was no difference in body length. (C) Increased food intake of *Hpa*-ko mice on high-fat diet but not on normal chow. *Hpa*-ko and wt control mice, age 10–15 weeks, were given either normal chow (*left* panel) or offered a choice between low-fat diet and high-fat diet during one week (*right panel*). No difference was seen between the two groups (wt, n = 6; *Hpa*-ko, n = 7) on normal chow. *Hpa*-ko mice (n = 7) ate significantly more of the high-fat diet (expressed as kcal/one week; p = 0.04) and more overall (p = 0.019) than wt controls (n = 5), whereas consumption of low-fat diet did not differ significantly between the groups.

Previous analysis of the heparanase knockout (*Hpa-*ko) mice showed neither remaining enzyme protein nor detectable catalytic activity in any of the tissues analysed [21]. The body weight of male *Hpa*-ko mice kept on normal chow was similar to that of *wt* siblings for the first 5–6 weeks of life but diverged thereafter. *Hpa-*ko mice weighed more than their *wt* siblings (Fig. 1A). *Hpa-*ko and congenic *wt* siblings were subjected to DEXA analysis of body composition at 21 weeks of age. Results showed that the difference in body weight was largely due to a substantial increase in fat mass in *Hpa-*ko mice, along with a smaller but significant increase in lean body mass (Fig. 1B). Notably, the fat content of the *Hpa*-ko lacking heparanase was almost twice that of *wt* controls. There was no difference in body length.

To investigate food consumption, individually housed adult male mice were given the choice between a low-fat and a high-fat diet for one week. *Hpa-*ko mice consumed significantly more of the high-fat diet and showed a higher overall energy intake than the *wt* controls (Fig. 1C, right panel). Neither the intake of low-fat diet (Fig. 1C, *right panel*) nor the intake of normal chow (with even lower fat content) when offered alone (Fig. 1C, *left panel*) differed between groups.

### Effects of heparanase overexpression on body composition, feeding response and energy metabolism

**Figure 2:**
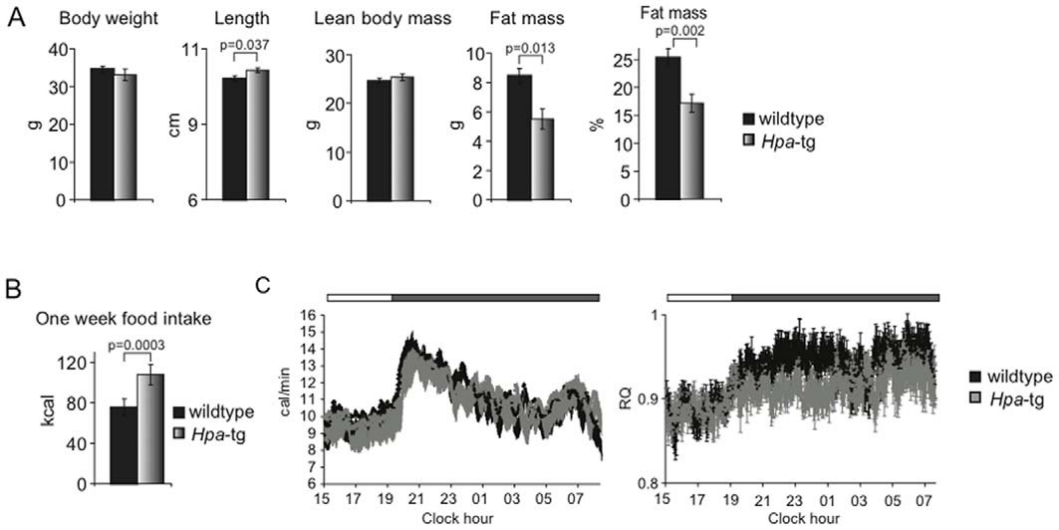
Reduced fat mass but increased food intake by *Hpa*-tg mice. (A) Body weight and body composition determined by DEXA analysis of *Hpa*-tg mice (n = 9) and wt controls (n = 8), age 12 weeks. No significant differences in lean body mass or body weight were detected, whereas the *Hpa*-tg mice showed reduced fat mass, and increased body length (nose-anus measurement). (B) Increased food intake by *Hpa*-tg (n = 5) compared to wt control mice (n = 4) during one week on normal chow. These animals were 36–37 weeks old, thus older than the mice tested in Fig. 1C. Mice were individually housed and weighed before and after the experiment. (C) Indirect calorimetry measurements on the animals (27 weeks old) showed no difference in energy expenditure (left panel) but a marked tendency toward lower RQ (right panel, p = 0.09 based on areas under the curves) for *Hpa*-tg mice.

Over-expression of heparanase in the *Hpg*-tg mice was confirmed immunocytochemically, with marked expression in many brain areas, including the hypothalamus (Fig. S1). The *Hpa-*tg mice showed a striking metabolic phenotype. DEXA analysis of *Hpa-*tg mice at 5 months of age revealed reduced fat mass, but no difference in lean body mass or body weight. Body length was significantly increased compared to *wt* controls (Fig. 2A). Unexpectedly, cumulative food intake monitored over a period of one week was significantly higher in the *Hpa-*tg group. Consumption of normal chow by individually housed animals was monitored once a day. *Hpa-*tg mice ate more chow than *wt* controls (Fig. 2B), indicating decreased food efficiency. Bomb calorimetry measurements revealed no difference in energy contents of faeces from *Hpa-*tg and *wt* animals (Fig. S2), hence excluding energy loss in faeces as a cause of the decreased food efficiency. However, analysis of energy metabolism by indirect calorimetry (Fig. 2C) showed that whereas *Hpa-*tg mice equalled *wt* energy expenditure, they shifted towards a lower RQ during the dark phase. The transgenic mice thus appear more prone to use fat as an energy source. Consistent with this metabolic phenotype, Q-PCR analysis of relevant genes showed only marginal effects of heparanase overexpression on *Synd-3* and *Pomc*, but significantly decreased hypothalamic expression of *AgRP*, compared to *wt* controls (Fig. S3).

### Intracerebroventricular injection of heparanase reduces food intake and body weight gain after fasting

**Figure 3:**
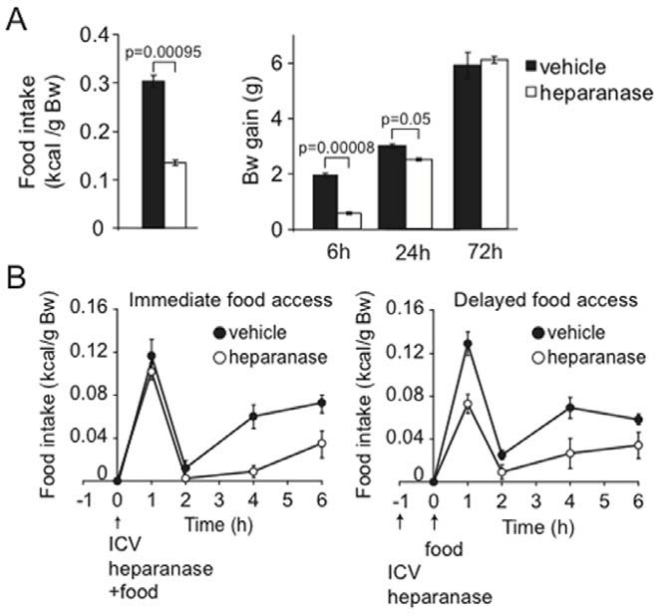
Intracerebroventricular injection of heparanase reduced food intake and body weight gain after fasting. (A) Effect on food intake. C57BL/6J mice (6–8 weeks old) with cannulae inserted for the ICV experiment were fasted for 16 hr, and were then subjected to ICV injection of recombinant heparanase as described in Methods. The mice were allowed access to normal chow immediately after injection, and food intake was measured over the subsequent 6 hr period. The heparanase-treated mice (n = 6) showed a >50% reduction in cumulative food intake, related to body weight, compared to controls (injection of vehicle; n = 6). The same mice were weighed at different time points following ICV injections. Reduction in body weight gain of heparanase injected mice, compared to controls, was most apparent after 6 hr, still discernible after 24 hr, but not detectable after 72 hr. (B) Time course of effects due to ICV heparanase injection. Four experimental groups were designed: (1) heparanase-injected mice (n = 4) and (2) vehicle-injected mice (n = 4) with immediate access to food (*left panel*); (3) heparanase-injected mice (n = 9) and (4) vehicle-injected mice (n = 5) given access to food 1 hr after the injection (*right panel*). Determination of chow consumption (expressed as kcal/g body weight) after the indicated time periods showed sustained reduction of food intake for groups (1) and (3) compared to the respective control groups (2) and (4). However, data restricted to the first hour of food access showed a significant difference only between groups (3) and (4) (p = 0.0046), not between groups (1) and (2) (p = 0.417).

According to the current hypothesis cell-surface HS promotes binding of AgRP to MC4R [13]. Truncation of these chains by heparanase would favour receptor binding of the agonist α-MSH and hence, cause a suppression of food intake. This hypothesis was tested in acute experiments involving injection of recombinant human heparanase into the lateral third dorsal ventricle along with a starvation-refeeding protocol. Intracerebro-ventricular (ICV) administration of heparanase caused a dramatic reduction in food intake compared to vehicle-treated control mice. Cumulative food intake at 6 hr after heparanase injection (and start of refeeding) was less than 50% of that observed in controls (Fig. 3A, *left panel*). This effect of heparanase injection was also evident in terms of body weight gain that was only 30% of control after 6 hr and still depressed after 24 hr (Fig. 3A, *right panel*).

Experiments designed to elucidate the time course of heparanase action revealed different temporal windows depending on when the enzyme was administered relative to the start of refeeding. When access to food was admitted immediately after heparanase injection, reflex hyperphagia was observed without any discernible difference between heparanase- and vehicle-treated groups during the first hour (Fig. 3B, *left panel*). Instead, the reduction in food intake developed gradually during the following hours, was most conspicuous 4–6 hr after enzyme administration, and persisted well beyond the time window required for control mice to resume normal feeding behaviour. Importantly, this finding suggests that heparanase affects food intake *per se* and not only in connection with reflex hyperphagia. We next investigated whether the delayed response was inherent to the mechanism triggered by the enzyme, alternatively, it could also be explained by pharmacokinetic factors. We therefore redesigned the experiment by administering the heparanase 1 hr prior to food presentation. Under these conditions the reduction in food intake was seen from the outset (Fig. 3B, *right panel*). The delayed action observed after simultaneous heparanase injection and food administration thus appears to reflect the time required for the enzyme to reach and act on the HSPG target.

**Figure 4:**
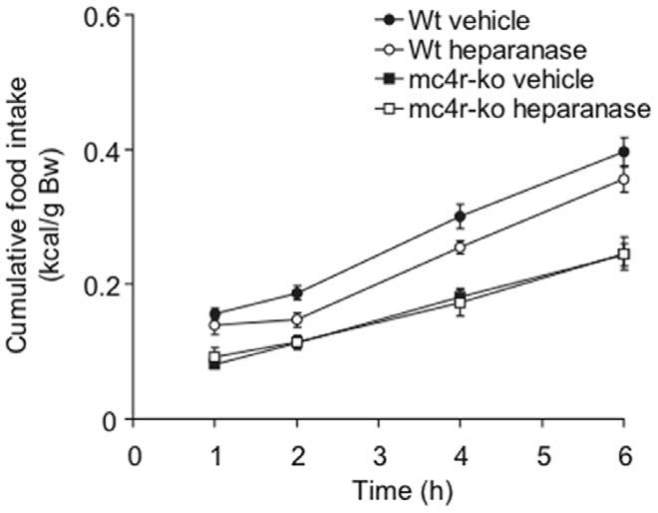
The acute effects of heparanase are mediated through the MC4R. *MC4r*-ko mice and wt siblings (6–8 weeks old) were subjected to the fasting-refeeding procedure, with ICV injection of 1.4 µg heparanase in a volume of 1 µl (n = 8 and n = 6, respectively) or vehicle (n = 6 and n = 7, respectively) 1 h before access to food. The heparanase injection of wt mice resulted in significant reduction in cumulative food intake, 2 hr (p = 0.023) and 4 hr (p = 0.0053) after refeeding, compared to vehicle-injected mice. Heparanase had no effect on the *MC4r*-ko mice.

Collectively, these results implicate heparanase in the modulation of MC4R signaling. To test this concept we designed a cross-over study in which food intake was measured in MC4R male knockouts and their *wt* siblings injected ICV with heparanase or vehicle. The results showed that *wt* mice injected with heparanase had 20–25% lower cumulative food intake 2 hr and 4 hr after food presentation compared to vehicle controls, whereas there was no effect of heparanase treatment in the MC4R knockout mice (Fig. 4). The acute effects of centrally administered heparanase on food intake can therefore be directly linked to modulation of MC4R signaling. We also infer that the appetite suppression in the *wt* mice was not due to general malaise/sickness induced by the heparanase injection.

## Discussion

### Heparanase is an important endogenous regulator of energy balance

Heparanase affects a variety of physiological and pathophysiological processes, essentially through cleavage of HS chains on HSPGs at cell surfaces or in the extracellular matrix [23]. Detailed analysis of the *Hpa*-tg mouse strain used in this study showed that cerebral expression of the enzyme led to degradation of HS, accompanied by change in disaccharide composition (X. Zhang, personal communication). Our results implicate heparanase with regulation of energy balance, through modulation of MC4R signaling. Male *Hpa-*ko mice display maturity-onset obesity and exhibit increased food intake when given access to a high-fat diet. Conversely, the *Hpg-*tg mice have reduced body fat. Moreover, central administration of heparanase lead to reduced food intake in mice and this effect was abolished in mice lacking MC4R.

The first observation associating a HSPG with appetite regulation was maturity-onset obesity in transgenic mice over-expressing syndecan-1 [16]. The constituent HS chains were assumed to promote binding of AgRP to its receptor, MC4R, thus stimulating food intake and at the same time precluding the appetite-suppressing effects of α-MSH. This co-receptor function would normally be exerted by syndecan-3, the predominant HSPG isoform in the hypothalamus. Notably, a polymorphism in the syndecan-3 gene has been associated with obesity in a female population [24]. The levels of syndecan-3 and hence, exposure of HS chains, at the cell surface, appear regulated through shedding of the HS-substituted ectodomain by protease cleavage and vary with the feeding state of the animal. It was proposed that the shedding enzyme that cleaves hypothalamic syndecan-3 does not recognize syndecan-1 [13], [16], [17], [18].

**Figure 5:**
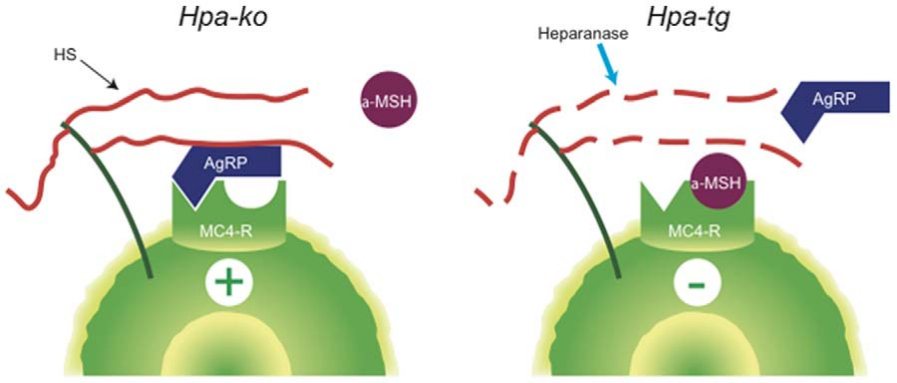
Proposed effects of heparanase on MC4R signaling. HS chains of a cell-surface HSPG serve as co-receptors for AgRP, and at the same time preclude access of α-MSH to the MC4R. *(Left panel)* In the absence of heparanase (*Hpa-*ko) the HS chains remain intact, resulting in continuous AgRP-induced activation of MC4R, thus promoting food consumption, fat accumulation, and a positive energy balance. Under these conditions α-MSH signaling is severely restricted. *(Right panel)* HS chains are degraded by overexpressed heparanase (*Hpa-*tg), thus rendering the MC4R accessible to α-MSH, whereas AgRP devoid of its HS co-receptor is unable to engage the MC4R and thus remains inactive. This setting explains the acute effects of ICV-injected heparanase that lead to transient yet drastic reduction in feeding. However, it is also compatible with the long-term effects of transgenic heparanase expression that shift energy balance toward increased loss of body fat and compensatory up-regulation of appetite.

Heparanase provides an alternative mechanism for modulating accessibility of HS chains. Our results indicate that heparanase affects energy balance, in particular, as a negative modulator of AgRP signaling, by cleavage of HS chains on the syndecan-3 co-receptor (Fig. 5). *Hpa*-ko mice thus develop a phenotype reminiscent of that observed in syndecan-1 over-expressing mice [16], with maturity-onset increase in body weight due to increased fat and, to a lesser extent, lean body mass (Figs. 1A, B). Regulation of food intake is affected as shown by increased intake of high-fat chow (Fig. 1C). Conversely, heparanase over-expressing *Hpa-*tg *mice* showed reduced fat mass, in seemingly logical contrast to the *Hpa*-ko phenotype (Fig. 2A). The predicted mechanism was corroborated by acute injections of heparanase directly into the lateral cerebral ventricle, in connection with fasting-refeeding experiments. This treatment resulted in a marked, sustained suppression of food intake (Fig. 3) that was dependent upon intact MC4R function (Fig. 4). Thus, the increased food intake of *Hpa-*ko mice may be ascribed to more efficient AgRP co-receptor function of HS chains (Fig. 5).

### Heparanase is involved in multiple facets of energy balance regulation

The MC4R signaling system is known to modulate multiple factors in regulation of energy balance [20], and the seemingly unified picture outlined through the *Hpa-*ko and *Hpa-*tg models in Fig. 5 appears partly oversimplified. Heparanase affects a number of distinct factors in energy balance regulation. Key components of this equation are energy intake through feeding and energy expenditure through basal metabolism, physical activity and adaptive thermogenesis [2]. Unexpected effects of heparanase overexpression may be ascribed to the complex regulatory mechanisms behind this balance. *Hpa*-tg males thus were unexpectedly found to eat more than *wt* controls (Fig. 2B), in spite of their reduced fat mass (Fig. 2A). Whereas there was no significant difference in energy expenditure, the *Hpa-*tg mice showed a lower nominal RQ indicating a relative preference for fat as the main fuel source (Fig. 2C). The decreased *AgRP* expression of the *Hpa-*tg mice (Fig. S3) is of particular significance in this context, as central melanocortin signaling also controls peripheral nutrition partitioning that may affect the amount of body fat independently of food intake [25]. In fact, *AgRP-*ko mice, a predicted phenotype mimic of our *Hpa*-tg animals (see Fig. 5) showed increased fat utilization [11]. The *Hpa*-tg mice appear more aggressive at adult age than *wt* controls but otherwise show no apparent deviation in behaviour. Overall, our data indicate that heparanase affects food intake primarily in a short-term perspective and energy balance over a longer time span. It thus has been suggested that AgRP acts both as an antagonist of the MC4R and as an inverse agonist on its own, and that the different modes of signaling relate to short-term effects of fasting and more long-term effects on energy balance regulation, respectively [17]. We therefore surmise that the increased food intake by the *Hpa-*tg mice is a compensatory phenomenon, secondary to the increased use of fat as energy source [26], [27].

The various phenotypes resulting from genetic perturbation of the melanocortin signaling system (Fig. 5) largely conform to our hypothesis that heparanase modulates energy balance *via* the melanocortin system, by compromising the AgRP—syndecan-3 interaction at the MC4R. The obese phenotypes seen in mice lacking MC4R [8] or over-expressing either AgRP or syndecan-1 [10], [11], [16] resemble that of *Hpa-*ko mice. Both the MC4R knockouts [28], [29] and heparanase knockouts (Fig. 1C) react by increasing food intake when challenged with a high-fat diet. Conversely, the syndecan-3 knockout and AgRP-deficient mice display lean phenotypes [11], [17] similar to the *Hpa-*tg mice.

The *Hpa*-tg mice used in this study differ from those described in a previous report, characterized by significantly reduced food intake and body weight compared to controls [22]. However, subsequent examination of these mice revealed a defective intestinal barrier and leakage of nutrients in the intestine [30]. Intestinal malfunction with nutrient leakage presumably confounds metabolic phenotype analysis. The mice used in the present work were from a different founder derived from the same construct [22], and were backcrossed to yield a pure genetic background (C57BL/6J). They had no apparent intestinal problems, most likely due to the low level of overexpression of heparanase in the intestinal system. However, this mouse strain showed more prominent heparanase expression in the brain (Fig. S1).

Human genetic data and murine studies have implicated MC4R with energy balance regulation in the CNS, hence a potential target for treatment of obesity and eating disorders [31], [32], [33]. Mutations in the MC4R are the most common cause of monogenic childhood obesity and the severity of obesity can be quantitatively correlated to the impairment of signaling [12], [34]. Moreover, several polymorphisms close to the MC4R gene were associated with BMI in recent genome-wide association studies [35], [36], [37]. The receptor apparently has a key role in coordinating afferent messages from the periphery and regulatory signals in control of food intake as well as energy expenditure. Our data indicate that heparanase acts as a negative modulator of AgRP signaling at MC4R, through cleavage of HS chains that are constituents of syndecan-3. This mechanism, along with previously proposed, modulated shedding of syndecan-3 ectodomain [13], [16], [17], [18], constitutes a novel signaling system that will help improve our understanding of obesity and its causes.

## Materials and Methods

### Mice

Male mice were used throughout the study. Heparanase overexpressing (*Hpa-*tg) mice were essentially as described in [22], except that the mice used in the present study were from a different founder and were backcrossed (×10) to C57BL/6 background. Heparanase cDNA was expressed under the chicken ß-actin promoter with a CMV-IE enhancer, followed by a rabbit ß-globin poly-adenylation site. The *Hpa-*ko mice were described in [21]. The genotypes of the mice were determined by PCR as described [21], [22]. *MC4R-*ko (B6;129S4-Mc4r_tm1Lowl_/J) mice [38] were purchased from The Jackson Laboratories (Maine, USA) and were further bred and raised at the local animal facility (Gothenburg University). For genotyping a three-primer touchdown PCR was used (Scandinavian Gene Synthesis AB, Köping, Sweden). All *MC4R-*ko mice were of mixed background (C57BL/129SV) and we therefore used only siblings as controls. C57BL/6J mice were purchased from Taconic, Denmark.

Mice were maintained on a 12-hour light, 12-hour dark schedule (lights on at 7 am). Food and water were available ad libitum except where otherwise indicated. Standard protocols of laboratory animal care were followed. The age of mice is indicated in connection with description of experiments. The experiments were approved by the ethical committee at Gothenburg University, Sweden (ID numbers: 211/08, 308/06 and 66/07).

### Genotyping

Genomic DNA was isolated from tail biopsies and was purified using “HotSHOT” genomic DNA preparation [39]. Primers for *Hpa-*ko: Neo U-5′-gaggctattcggctatgactg-3′ and Neo L-5′-aggagcaaggtgagatgaca-3′; *Hspe* U-5′-cgttcctgtccatcaccatc-3′ and *Hspe* L-5′- aggctccagacaaagtgctaa-3′; For *Hpa-*tg: Primers were as in [22]. For *MC4R-*ko: common MC4r#1 5′-gcagtacagcgagtctcagg -3′, mutant MC4r#2 5′-gtgcaagtgcaggtgccag-3′ and wild type MC4r#3 5′-ctccaacaggcttatgacacc-3′, yielding a band of 500 bp (mutant) and a band of 400 bp (wildtype).

### Analysis of protein and gene expression

For gene expression, the brains of the mice (12–18 weeks old) were dissected and total RNA was extracted from hypothalamus. Q-PCR was applied using the primers shown in Table S1. The data were normalized using the mRNA level of GAPDH from each sample. For immunohistochemical staining, the brain sections were stained with anti-heparanase antibody (733) and counter-stained with H&M as described previously [22].

### Diets and food consumption

High-fat diet, HFD (D12492; 60% fat, 20% protein, 20% carbohydrate by calories, 5.24 kcal/g), and low-fat diet, LFD (12450B; 10% fat, 20% protein, 70% carbohydrate by calories, 3.85 kcal/g) were purchased from Research Diets Inc. (New Brunswick, USA). Normal chow (4% fat, 16.5% protein, 58% carbohydrate by calories, 2.99 kcal/g) was provided by the animal facility for routine maintenance (product number R34; Lantmännen, Stockholm, Sweden). Food intake was measured manually, involving weighing of pre-weighed food pellets at times indicated.

### Intracerebroventricular heparanase injections

Recombinant heparanase for injection experiments was generated using single-chain GS3 active heparanase gene construct, comprising the 8 and 50 kDa heparanase heterodimer (8+50), kindly provided by Dr. Christian Steinkuhler (IRMB/Merck Research Laboratories, Pomezia, Italy). The recombinant protein was purified to homogeneity (crystallization grade) from the conditioned medium of baculovirus-infected cells [40]. GS3 active heparanase was assayed for the presence of bacterial endotoxin by Biological Industries, using the gel-clot technique (Limulus amebocyte lysate, LAL test) and was found to contain <10 pg/ml endotoxin [41].

For ICV surgery, mice (6–8 weeks old; C57BL/6J) were anaesthetized using isofluran (induction 4%, maintenance 2–3%; air flow, 260 ml/min) and then placed in a stereotactic frame. Holes through the skull were made using a dentist drill, one for a steel guide cannula (AG-8, code 806302 from Agntho's, Lidingö, Sweden) that was implanted projecting to the lateral ventricle using stereotactic co-ordinates (0.94 mm posteriorly from bregma and down 1 mm) and one for a stabilizing screw (Agntho's). The cannulae were held in position by dental cement (Dentalon, Agntho's, fluid 7509, powder 7508) attached to the stabilizing screw. Saline (approximately 500 µL/mouse) was given subcutaneously for hydration and Rimadyl (2.5 mg/kg mouse) for analgesic was administered intraperitoneally. After 5–6 days of recovery on normal chow in individual cages the mice were fasted for 16 h over-night before ICV injections. Food consumption and weight regain were recorded as described in the results.

Recombinant heparanase (1.4 µg/µL) in vehicle (20 mM MES, 0.15 M NaCl, pH 6.0) was injected (1 µL/min, total 1 µL) using CMA pumps (CMA Microdialysis AB, Solna, Sweden) and Hamilton syringes (Genentec, Västra Frölunda, Sweden) connected to injection cannulae (30G, AMI-9.5; Agntho's). The vehicle was used as a control. Food intake was measured at the time points indicated. To check placement of needle, trypan blue was injected into the guide cannulae after the experiment followed by dissection of the brains.

### Energy consumption, respiratory quotient and body composition

Mice at varied ages were used in the different experiments (see legends to figures); however, *wt* and transgenic mice in each experiment were invariably of similar age. Indirect calorimetry was performed using the INCA systems (Somedic, Hörby, Sweden). Mice were placed individually in metabolic chambers 2 hr before start of measurement for adaptation, and were then maintained in the chambers for 16–20 hr with ad libitum access to food and water. Oxygen consumption and carbon dioxide release were measured at 2 min intervals. Data were analysed by calculating areas under curves followed by two-tailed independent t-tests. Body composition was determined by dual energy X-ray absorptiometry (DEXA) using a Lunar PIXImus (GE Lunar, Madison, WI, USA). Body length was measured on sedated mice with a ruler from nose to anus.

### Statistical procedures

Data were analysed using independent two-tailed t-test and two-way analysis of variance (ANOVA) using the SPSS program (SPSS, Chicago, IL, USA). All data presented are means ± SEM. P values reported are from independent two-tailed t-tests unless otherwise stated.

## Supporting Information

Figure S1 Immunohistochemical display of human heparanase expression in *Hpa-*tg mouse brain. Brain sections were stained with anti-heparanase antibody (733; red). The heparanase positive cells in the hypothalamic nucleus are indicated by *arrows*.
(TIF)

Figure S2 Faecal energy contents determined by bomb calorimetry. Faeces was collected from *wt* (n = 4) and *Hpa-*tg (n = 5) mice (16 months old) and submitted to bomb calorimetry, (C 5000, IKA®Werke GmbH & Co. KG, Germany).
(TIFF)

Figure S3 Expression of *Synd3*, *Pomc* and *AgRP* genes in hypothalamus.The brains of mice (24–28 weeks old) were dissected, and RNA was extracted from the hypothalamus and analyzed for gene expression by Q-PCR, as described in Methods.
(TIF)

Table S1 Primers used in quantitative PCR.
(DOCX)
