# Correction: Heparanase Affects Food Intake and Regulates Energy Balance in Mice

**DOI:** 10.1371/annotation/c91d94c7-fd12-4a77-ba18-d3ff4f163d72

**Published:** 2012-10-26

**Authors:** Linda Karlsson-Lindahl, Linnéa Schmidt, David Haage, Caroline Hansson, Magdalena Taube, Emil Egeciouglu, Ying-xia Tan, Therese Admyre, John-Olov Jansson, Israel Vlodavsky, Jin-Ping Li, Ulf Lindahl, Suzanne L. Dickson

The sixth author name was spelled incorrectly. The correct name is: Emil Egecioglu. 

