# Supplementary figures and images for: Heparanase Affects Food Intake and Regulates Energy Balance in Mice
Source: PLoS One. 2012 Mar 27;7(3):e34313. doi: 10.1371/journal.pone.0034313 (PMC3313980; doi:10.1371/journal.pone.0034313)

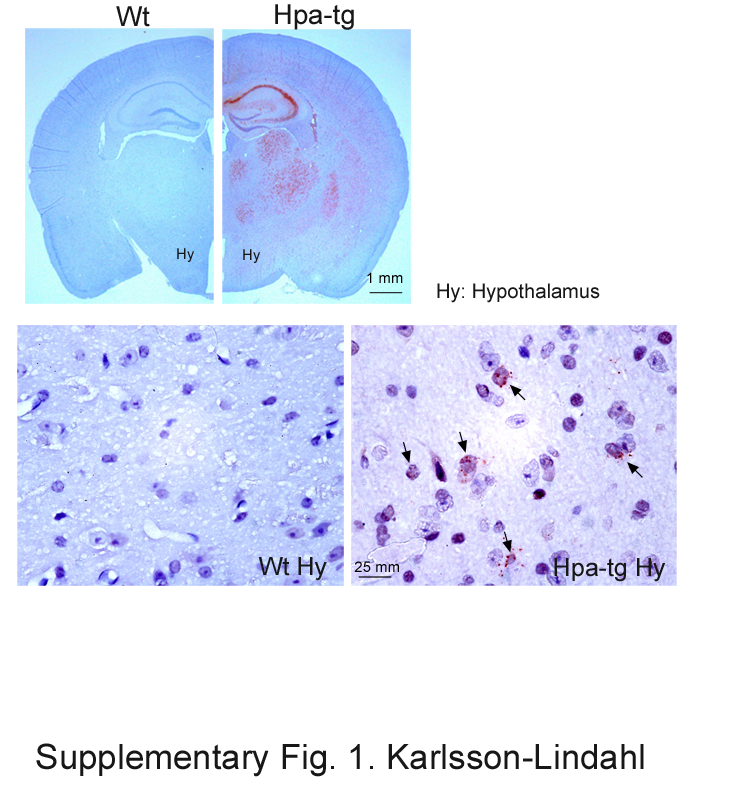

Supplement: Figure S1 — Immunohistochemical display of human heparanase expression in Hpa-tg mouse brain. Brain sections were stained with anti-heparanase antibody (733; red). The heparanase positive cells in the hypothalamic nucleus are indicated by arrows. (TIF) [file pone.0034313.s001.tif]

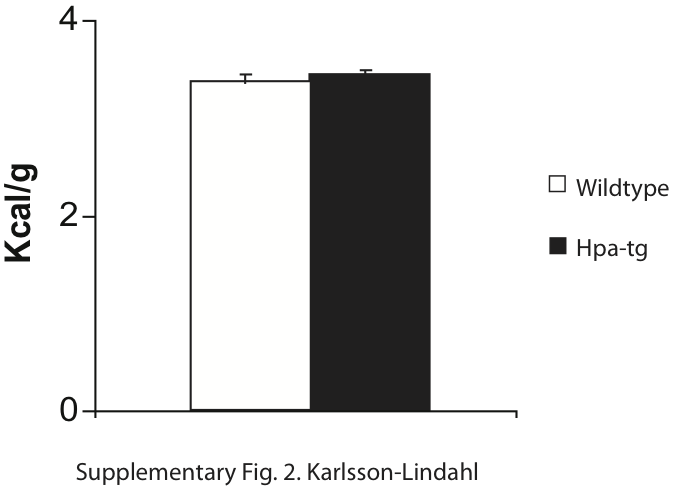

Supplement: Figure S2 — Faecal energy contents determined by bomb calorimetry. Faeces was collected from wt (n = 4) and Hpa-tg (n = 5) mice (16 months old) and submitted to bomb calorimetry, (C 5000, IKA®Werke GmbH & Co. KG, Germany). (TIFF) [file pone.0034313.s002.tiff]

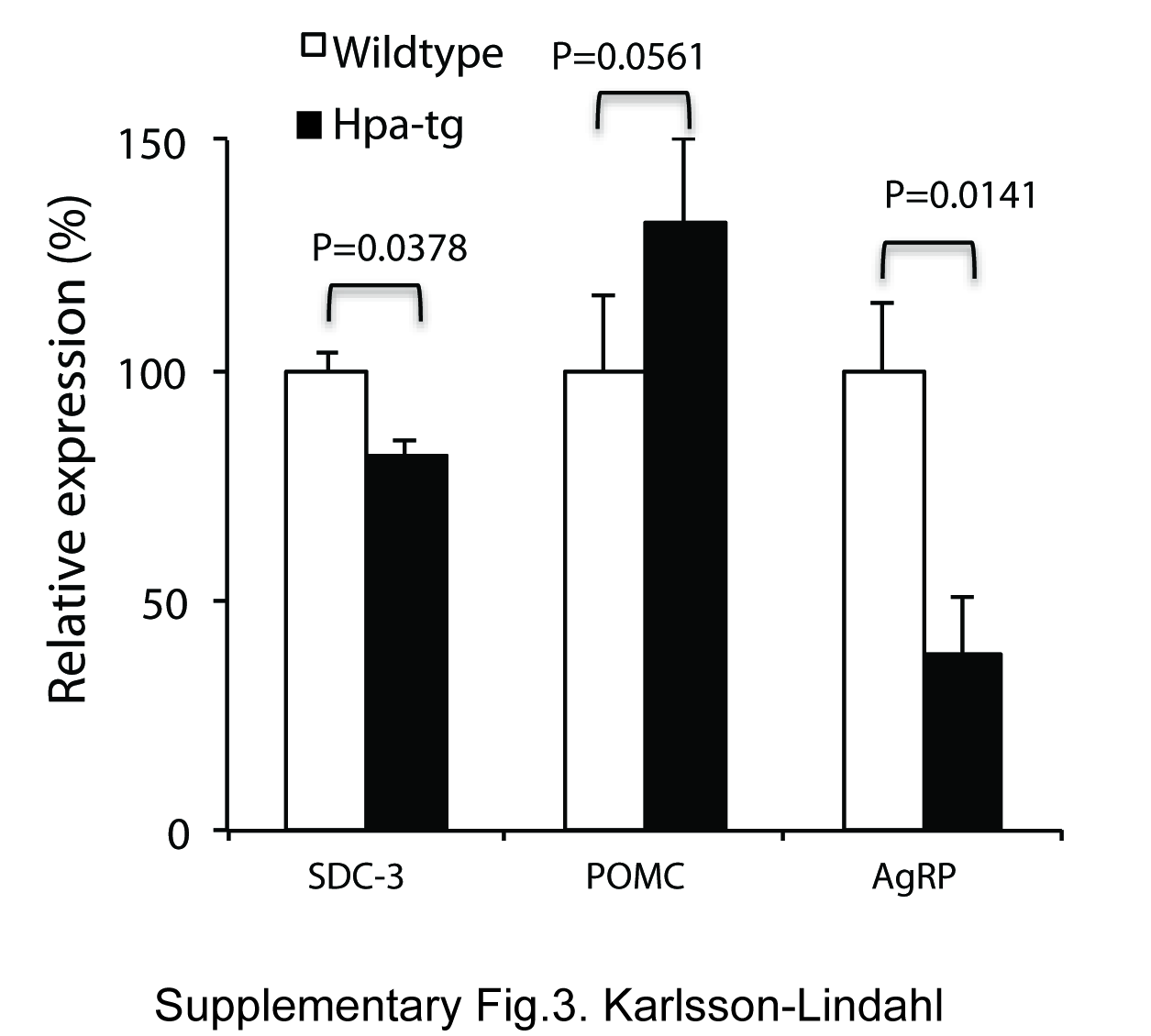

Supplement: Figure S3 — Expression of Synd3, Pomc and AgRP genes in hypothalamus.The brains of mice (24–28 weeks old) were dissected, and RNA was extracted from the hypothalamus and analyzed for gene expression by Q-PCR, as described in Methods. (TIF) [file pone.0034313.s003.tif]
