# Supplementary material for: Heparanase Affects Food Intake and Regulates Energy Balance in Mice
Source: PLoS One. 2012 Mar 27;7(3):e34313. doi: 10.1371/journal.pone.0034313 (PMC3313980; doi:10.1371/journal.pone.0034313)
Supplement: Table S1 — Primers used in quantitative PCR. (DOCX) [file pone.0034313.s004.docx]

| **Gene** | **Forward primer (5’ 3’)** | **Reverse primer (5’ 3’)** |
| --- | --- | --- |
| m*POMC* | GTGCCAGGACCTCACCAC | CTTCCGGGGGTTTTCAGT |
| m*AgRP* | CTCAAGAAGACAACTGCAGAC | TGAAGAAGCGGCAGTAGCAC |
| m*Sdc-3* | ATACTGGAGCGGAAGGAGGT | TTCATGCGGTAGATGAGCAG |
| m*GAPDH* | ACTCCACTCACGGCAAATTC | TCTCCATGGTGGTGAAGACA |

**TABLE 1.** Primers used in quantitative PCR
